# Boosting Antioxidant Quality in Cucumber Beverages with Encapsulated Tomato Carotenoids

**DOI:** 10.3390/antiox14030354

**Published:** 2025-03-18

**Authors:** Laleh Mozafari, Lorena Martínez-Zamora, Marina Cano-Lamadrid, Perla A. Gómez, Francisco Artés-Hernández

**Affiliations:** 1 Postharvest and Refrigeration Group, Department of Agricultural Engineering and Institute of Plant Biotechnology, Universidad Politécnica de Cartagena, 30203 Cartagena, Murcia, Spain; laleh.mozafari@edu.upct.es (L.M.); lorena.martinez@upct.es (L.M.-Z.); perla.gomez@upct.es (P.A.G.); 2 Department of Food Technology, Food Science and Nutrition, Faculty of Veterinary Sciences, University of Murcia, 30071 Murcia, Spain

**Keywords:** bioactive compounds, tomato by-products, revalorization, encapsulation, cucumber (*Cucumis sativus*) juice, high hydrostatic pressure (HHP)

## Abstract

Tomato by-products are widely generated during processing, which deserve revalorization due to being rich in bioactive compounds that can be incorporated into novel formulas. This study explores the use of tomato by-products as a source of pigments and antioxidant compounds to develop a seasoned cucumber beverage enriched with encapsulated carotenoids. Extracts from industrial tomato pomace were obtained using ultrasound-assisted extraction (USAE) and accelerated solvent extraction (ASE), and then encapsulated by spray-drying with inulin (I), maltodextrin (M), or a maltodextrin–inulin blend (MI). The powders were added to a cucumber beverage treated with high hydrostatic pressure (HHP) and stored for 28 days at 4 °C. Physicochemical properties, microbial load, carotenoid content (U-HPLC), free phenolic content (FPC), and total antioxidant capacity (TAC) were monitored. Beverage samples with maltodextrin (ASE-M, USAE-M) and the maltodextrin–inulin blend (ASE-MI, USAE-MI) showed superior color stability and pH maintenance. USAE-MI achieved the highest TAC at the end of storage and ensured microbial safety by reducing mesophilic bacteria, molds, and yeast. During storage, FPC declined (to ~3.5–5 mg 100 mL^−1^), TAC increased (to ~16–20 mg 100 mL^−1^), and carotenoid was kept stable (~9–13 mg L^−1^). These results highlight the potential of combining HHP with tomato by-product encapsulates to improve the shelf life, quality, pigment stability, and antioxidant properties of vegetable-based beverages.

## 1. Introduction

According to the Food and Agriculture Organization, approximately $400 billion per year worth of fruits and vegetables (F&V) is reportedly lost before reaching grocery stores [1]. A portion of these losses includes peels, seeds, rinds, husks, rags, roots, and pomace, among other non-edible parts of F&V [2]. Particularly, tomato pomace made of peels and seeds is obtained from the production of tomato derivatives such as fried tomato, sauces, or grated tomato and is currently used as a fertilizer and animal feed [3,4]. Nevertheless, these by-products contain significant amounts of bioactive compounds that can be useful as functional ingredients with antioxidant properties but can also damage the ecosystem and the environment due to their high biological oxygen demand [5].

In this context, the natural abundance of dietary antioxidants and pigments in tomatoes, such as carotenoids—primarily *β*-carotene and lycopene—has been associated with a reduced risk of cardiovascular disease and certain types of cancer [6,7]. To meet consumer demand for natural, safe, and healthy foods, researchers are exploring the use of extracted fruit and vegetable by-products as novel ingredients, with preservative effects already demonstrated in various food matrices [2,4], leading to improved nutritional properties.

The extraction and isolation of these bioactive compounds with antioxidant properties have been developed in the last years to become greener and more sustainable by reducing energy consumption and the amount of solvent used, substituting them with non-pollutant extractant agents. Among novel technologies, ultrasound-assisted extraction (USAE) and accelerated solvent extraction (ASE) have been developed as promising methods to revalorize food by-products. Particularly, ASE offers many benefits by reducing the operation time, enhancing extraction yields through elevated temperatures and pressures, decreasing solvent usage, and improving the stability of degradable compounds throughout the extraction process by maintaining samples in an environment devoid of oxygen and light [8,9]. On the other hand, the application of ultrasound technology to enhance the extraction of phytochemicals has also proven effective in reducing the processing time, energy consumption, and solvent use while increasing the bioavailability of the extracted compounds [10,11].

Once these carotenoids have been extracted, encapsulation can be beneficial to prevent degradation caused by their sensitivity to external agents and to ensure their stability, water solubility, and high bioavailability when these compounds reach the intestine for absorption by the human body [12,13]. In this regard, the polysaccharides constituting the core and protective matrix materials with antioxidant abilities, which combine to form the structures during the encapsulation process, may also enhance the prebiotic functionality of the resulting formulation [13]. For instance, maltodextrin is a hydrolyzed starch made by partially hydrolyzing starch with acid or enzymes [14,15], while inulin, as fructooligosaccharide (FOS), is commonly used as a low-calorie sweetener, texturizer, and gelling agent [16,17].

Furthermore, the global green juice market has grown exponentially due to increased health and wellness awareness. North America leads with high health consciousness and disposable income, while Europe favors organic products. In this context, cucumber-based drinks are gaining popularity worldwide. Known for its mild, refreshing taste, cucumber is often used in both culinary and wellness products. These beverages are low in calories and provide a natural hydration boost, making them a healthy choice for many consumers [18,19].

To ensure the quality and safety of these new formulations enriched in bioactive compounds as antioxidants, either encapsulated or not, the application of non-thermal processing techniques has been developed to reduce the microbial load while maintaining the sensory and nutritional quality [20]. This has led to increasing interest in high hydrostatic pressure (HHP), which inactivates microorganisms while not breaking down compounds responsible for sensory and nutritional properties, without increasing the process temperature (~35 °C), preserving the freshness and antioxidant value of the product [21]. This technology has been widely applied to preserve the quality of F&V-based products, even in reformulated products enriched in by-product extracts. The main novelty of the present work is based on the combination of different extraction and encapsulation techniques to obtain powders used to fortify a seasoned cucumber beverage treated under 400 MPa HHP, which was subjected to a shelf-life study. Therefore, the objective of the present work is to evaluate the effectiveness of six different encapsulated tomato by-products previously characterized [22] in an aqueous food matrix, assessing their ability to preserve the quality of this vegetable-based beverage over 28 days of storage at 4 °C.

## 2. Materials and Methods

### 2.1. Obtention and Encapsulation of Antioxidant Tomato By-Product

By-products from the industrial processing of tomato sauce (*Red Sky* pear tomato *cv*.) were sourced from Hida Alimentación (Murcia, Spain) and transported to the facilities of the National Technological Centre for the Food and Canning Industry (CTNC), where the extractions (USAE and ASE) and encapsulation were carried out as previously described [22]. An ultrasonic processor (Hielscher UIP500hdT, Hielscher, Germany) featuring a 100 mm diameter, 500 W power, and operating at 20 kHz, was used for USAE with a 25-micron amplitude and fully adjustable amplitude settings at 100%. A Thermo Scientific™ Dionex™ ASE™ 350 Accelerated Solvent Extractor (Thermo Scientific, Sunnyvale, CA, USA) was utilized for ASE, as previously specified [22]. The hydrophilic mix obtained was homogenized with the carrier selected (inulin: I, maltodextrin: M, and 1 maltodextrin/1 inulin: MI) and atomized at a rate of 6 mL min^−1^ in a mini spray dryer (Buchi S300, Flawil, Switzerland) as previously described [22]. Inulin obtained from chicory was supplied by Trades S.A. (Tarragona, Spain) and maltodextrin was produced by Amylum Slovakia spol s.r.o (Boleràz, Slovakia) and supplied by Brenntag Quimica S.A.U. (Sevilla, Spain). The characterization of the capsules and their content in bioactive compounds with antioxidant properties has been previously described [22]. The encapsulates were kept at −18 °C until their incorporation into the vegetable-based formulas.

### 2.2. Reformulation of the Seasoned Cucumber Beverage

**Figure 1:**
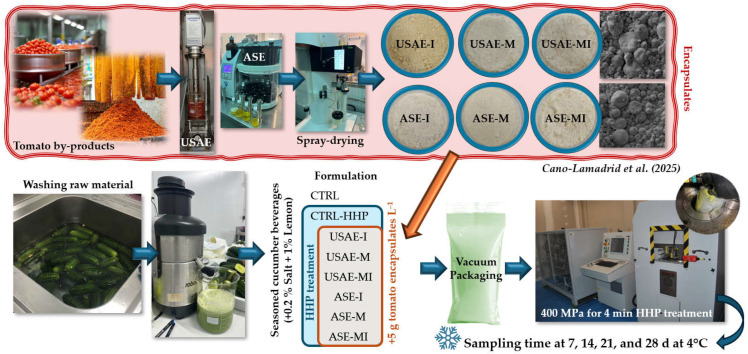
Experimental design used for the reformulation of the seasoned cucumber beverage fortified with different encapsulates of tomato by-products described by Cano-Lamadrid et al. [22].

Cucumbers were bought at a local supermarket and transported to the pilot plant of the Institute of Plant Biotechnology at Universidad Politécnica de Cartagena. The average quality parameters were L*: 32.1 ± 2.0; a*: −10.0 ± 1.4; b*: 14.7 ± 2.5; equatorial caliber: 49.1 ± 4.1 mm; longitudinal caliber: 124.6 ± 6.3 mm; and firmness: 131.2 ± 20.7 N. After disinfection (100 mg L^−1^ free chlorine at pH 6.5 for 2 min) and rinsing with cold water (5 °C), the juice was freshly squeezed using a food processor (Robot Cook^®^, Robot Coupe; Vincennes, Île-de-France, France). Three control formulas without encapsulates of tomato by-products were elaborated to compare the effect of the seasoning and the processing treatment. CTRL was the seasoned beverage with 0.2% salt and 1% lemon juice, while CTRL-HHP was the seasoned beverage with a treatment of 400 MPa for 4 min using a high-pressure Iso-Lab system (Stansted Fluid Power Ltd., Harlow, UK), as recommended by previous authors [23]. Once the recipe and the HHP were selected, six different formulas were prepared by their fortification with 5 g kg^−1^ encapsulated tomato by-products with different extractions (USAE and ASE) and different carriers (I, M, and MI), being USAE-I, USAE-M, USAE-MI, ASE-I, ASE-M, and ASE-MI samples. A total of 70 mL of samples were vacuum packaged into aseptic polypropylene bags (Infantine, San Diego, CA, USA). After elaboration of the different samples, an HHP processing treatment was applied, whose conditions are shown in Figure S1. Then, 6 bags (3 replicates for microbial and 3 for the rest of the analysis) per sample and sampling day (0, 7, 14, 21, and 28) were stored at 4 °C (*n* = 240), and the temperature was monitored using a Tinytag ULTRA 2 data loggers (TGU-4500; Chichester, UK). The experimental scheme of the present experiment is shown in Figure 1.

### 2.3. Microbial Analysis

The growth of mesophilic and psychrophilic bacteria, enterobacteria, molds, and yeasts was evaluated following standardized methods [24]. Plate count agar (PCA) was used as the culture medium for mesophiles and incubated for 3 days at 30 °C. Rose bengal agar was incubated for 7 days at 22 °C for molds and yeasts, and violet-red bile glucose agar was employed for enterobacteria and was incubated for 2 days at 37 °C. Microbial counts were reported as log colony-forming units per mL of fresh product (log CFU mL^−1^). Each sampling day involved the analysis of three replicates and three dilutions per sample.

### 2.4. Physicochemical Analysis

Density was measured after elaboration using a pycnometer, following standardized methods [25]. Color (L*, a*, and b*), pH, and total soluble solids were monitored every sampling day. A colorimeter (Konica Minolta CR-400) was used to determine the color coordinates, and variations (ΔE) throughout refrigerated storage compared to initial values were calculated according to the formula: ΔE = [ΔL*^2^ + Δa*^2^ + Δb*^2^]^1/2^. For pH analysis, a GLP21 pH-meter (Crison; Alella, Cataluña, Spain) was used. A digital hand-held refractometer (Atago N1; Tokyo, Kanto, Japan) was used to measure total soluble solids (TSS), expressed as g sugar equivalents 100 mL^−1^. All analyses were carried out in triplicate.

### 2.5. Extraction and Analysis of Main Bioactive Compounds with Antioxidant Properties

#### 2.5.1. Free Phenolic Content and Total Antioxidant Capacity

The free phenolic content (FPC) and total antioxidant capacity (TAC) were spectrophotometrically evaluated following the methods of Folin–Ciocalteu [26] for FPC and the ferric reducing antioxidant power (FRAP) assay [27] for TAC. For this, a 1:3 (sample-to-solvent ratio) 80% methanolic extraction was carried out for 1 h at 1× *g* and 4 °C for every sample. After centrifugation, the supernatant was collected and used for further analysis. For FPC, 19 μL of the extract was mixed with 29 μL of 1 N Folin–Ciocalteu reagent in a 96-well plate, followed by 192 μL of 0.4% Na_2_CO_3_ and 2% NaOH solution. After 1 h under dark conditions, absorbance was recorded at 750 nm in a microplate reader (Infinite PRO 2000; Tecan Trading AG, Männedorf, Switzerland), and results were expressed as mg of gallic acid equivalents (GAE) per 100 mL (y = 0.0936x − 0.0036; R^2^ = 0.9824). For TAC by FRAP, the absorbance was measured at 495 nm after 30 min of incubation in darkness with 6 μL of extract and 198 μL of daily FRAP solution (0.3 M sodium acetate buffer, pH = 3.6; 0.02 M FeCl_3_; 0.01 M tris 2-pyrigil-triazine (TPTZ); 10:1:1). Results were expressed as mg of Trolox equivalents (TE) per 100 mL (y = 0.4429x − 0.0404; R^2^ = 0.9711). All analyses were carried out in triplicate.

#### 2.5.2. Carotenoid Content

Carotenoid extraction, identification, and quantification were conducted in triplicate, following established protocols [28]. For the extraction process, 1 mL of cucumber beverage was combined with 4 mL of a chloroform–dichloromethane solution (2:1, *v*:*v*). The mixture was homogenized for 1 min using a standard homogenizer (Schott, Staufen, Germany) and then manually shaken for 30 s. Subsequently, it was subjected to orbital shaking (Stuart, Staffordshire, UK) at 1× *g* for 20 min at 4 °C. To achieve phase separation, 2 mL of NaCl solution was added. The sample was centrifuged at 3578× *g* for 10 min at 4 °C, and the organic layer was collected. The extraction process was repeated by adding 2.5 mL of chloroform–dichloromethane (2:1, *v*/*v*), followed by centrifugation, and the organic phase was retrieved again. The collected extracts were evaporated to remove solvents, reconstituted in 4 mL of a methanol–MTBE (40:60, *v*/*v*) solution, and filtered through a 0.2 μm PTFE membrane. The identification and quantification of carotenoids were carried out using an Ultra High-Performance Liquid Chromatography (UHPLC) system (Shimadzu, Kyoto, Japan), which was equipped with a C32 column, a DGU-20A degasser, an LC-30CE quaternary pump, a SIL-30AC autosampler, a CTO-10AS column heater, and an SPDM-20A photodiode array detector. The carotenoid profile, including lutein and *β*-carotene, was expressed as mg of carotene equivalents per L of sample. For that, the standard curve for *β*-carotene was y = 134170x + 2930.1; R^2^ = 0.9899; LOD: 0.056 mg L^−1^; LOQ: 0.1867 mg L^−1^. The electromagnetic spectrum showing the absorbance of such compounds is presented in Figure S2.

### 2.6. Statistical Analysis

A two-way (sample × storage time) analysis of variance (ANOVA) was performed to determine significant differences among samples and sampling time (*p* < 0.05) regarding parameters evaluated (*n* = 3). Statgraphics Plus software Centurion XV.II (v.5.1. Statpoint Technologies. Inc., Warrenton, VA, USA) was used for comparison among means with the Tukey test (95% confidence level). Correlation coefficients among obtained results of studied variables were calculated using RStudio software (v. 4.3.0 Posit, Boston, MA, USA).

## 3. Results

### 3.1. Physicochemical Evolution During Shelf Life

**Figure 2:**
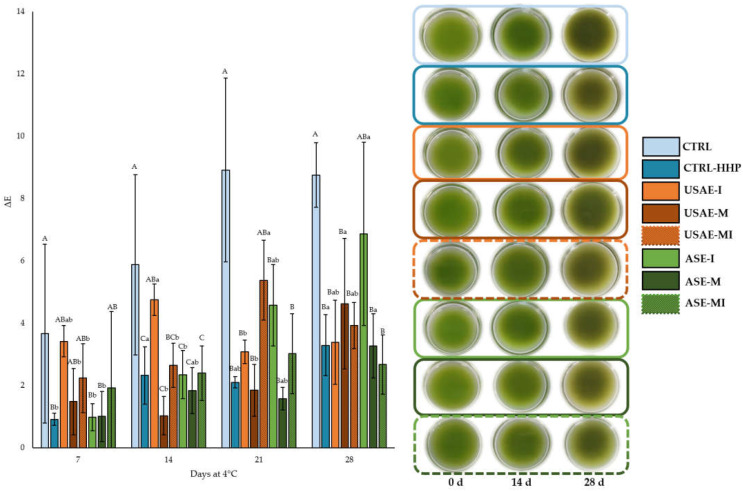
Color variations regarding initial values and visual appearance of seasoned cucumber beverages over 28 days at 4 °C (mean value of three replicates ± SD). Different capital letters denote significant differences among treatments (*p* < 0.05). Different lowercase letters denote significant differences among sampling days (*p* < 0.05). The absence of letters indicates no significant differences (*p* > 0.05) among treatments or sampling days.

The evolution of the physicochemical parameters is shown in Table 1, for pH, TSS, and most significant values of color: a*, b*, and chroma. Table S1 shows the obtained values of luminosity (L*) for the studied samples, while Figure 2 shows the visual appearance and the color changes during the refrigerated storage of seasoned cucumber beverages for 28 days at 4 °C.

Apart from these results, the density of the seasoned cucumber beverages after elaboration, measured with a pycnometer, was 1090.7 ± 16.4 kg m^−3^ without significant differences among the samples studied, which shows that the incorporation of the encapsulates of tomato by-product did not affect the density or viscosity of the samples.

As shown, the pH values of all the samples studied ranged from 4.60 to 4.87, with no significant differences among treatments till the second week of refrigerated storage, when the pH was slightly higher in USAE-M samples. In fact, these values are due to the inclusion of lemon juice in the seasoned cucumber beverage, as the pH values of the squeezed cucumber juice were from 5.40 at day 0 to 5.60 after 28 days at 4 °C. This fact justifies the inclusion of lemon, salt, and pepper to improve the sensory perception of the formula, as well as reducing the pH of this beverage to reduce the microbial load.

As anticipated, TSS levels were significantly higher in samples containing encapsulated tomato by-products, with values ranging from 4.7 to 5.6 g of sugar per 100 mL of juice. In contrast, the CTRL and CTRL-HHP samples exhibited TSS values between 4.2 and 4.7 g per 100 mL. This increase can be attributed to the use of inulin and maltodextrin as carrier agents in the encapsulation of tomato by-products. Specifically, these carriers, which form protective encapsulates to preserve the antioxidant compounds of tomato extracts, consist of fructo-oligosaccharides in the case of inulin and dextrose equivalents in the case of maltodextrin, thereby explaining the observed rise in TSS content in the encapsulated samples.

Regarding color evolution, the values showed for L*, a*, b*, and chroma ranged from 33.6 to 36.7 for L* (Table S1), from −8.2 to −4.9 for a*, from 7.4 to 9.7 for b*, and from 9.3 to 12.6 for chroma (Table 1).

These values, together with the representation of color variations and visual appearance of seasoned cucumber beverages (Figure 2), demonstrated that the end of the shelf life was established at 4 weeks after elaboration due to the presence of brown tones of the natural light green color of the cucumber beverage. In fact, important color changes were observed in the CTRL sample (from 6 to 10) due to the absence of the 4 min 400 MPa HHP treatment, which reported the browning characterized by the slight increase in a* simultaneously to the decrease in b* just after 2 weeks of refrigerated storage. By contrast, all HHP-treated samples showed color variations from 1–3 after 7 days of storage to 3–5 after 28 days, except for the ASE-I sample, which did not show a difference compared to CTRL on that sampling day.

### 3.2. Microbial Load During Shelf Life

**Figure 3:**
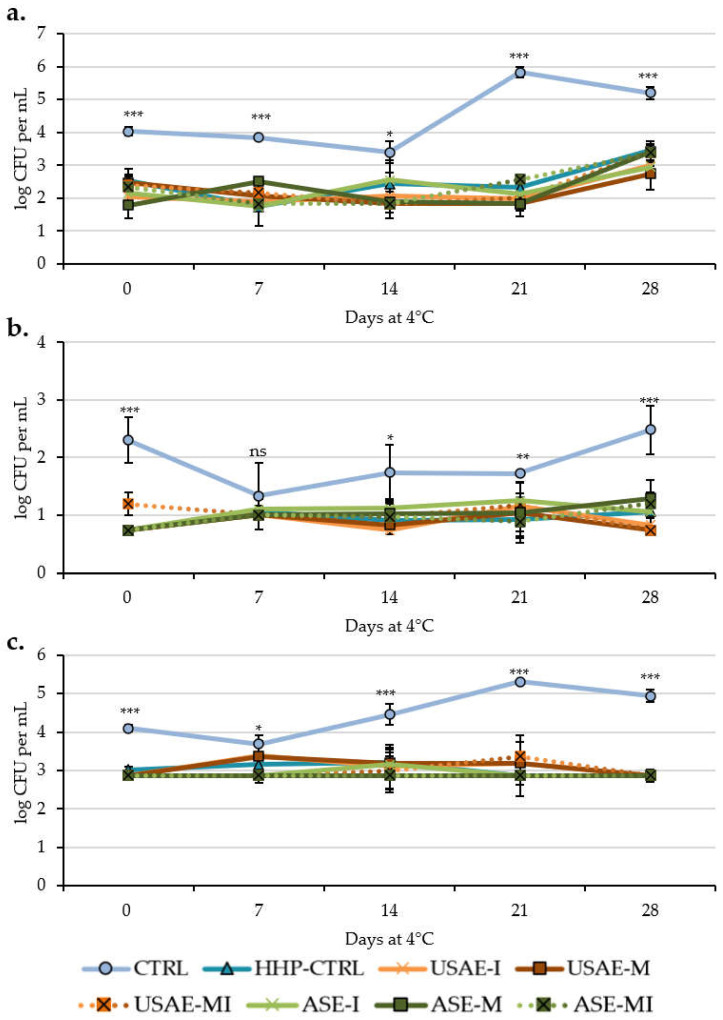
Mesophilic (**a**), *Enterobacteriaceae* (**b**), and molds and yeasts (**c**) growth in seasoned cucumber beverages over 28 days at 4 °C (mean value of three replicates ± SD). *: *p* < 0.05; **: *p* < 0.005; ***: *p* < 0.001; ns: *p* > 0.05. Detailed ANOVA results are shown in Table S2, in which (i) different capital letters denote significant differences among treatments (*p* < 0.05); (ii) different lowercase letters denote significant differences among sampling days (*p* < 0.05); (iii) the absence of letters indicates no significant differences (*p* > 0.05) among treatments or sampling days.

The effects of the HHP treatment and the application of encapsulated tomato by-products on the microbial load of seasoned cucumber beverages are shown in Figure 3. As shown, the application of a 4 min 400 MPa HHP treatment in reformulated samples showed a decrease of ~1.5–2 log CFU mesophiles and *Enterobacteriaceae* per mL, and ~1 log CFU molds and yeasts per mL of sample in comparison with CTRL sample. These reductions are widely appreciated also after 28 days of refrigerated storage in all studied samples, reaching values of microbial reduction by ~47% for mesophiles, ~70% for *Enterobacteriaceae*, and ~23% for molds and yeasts (a reduction by ~2–2.5 log CFU per mL of sample) in comparison with the CTRL cucumber beverage. As shown in the table within Figure 3, which presents the significant differences among the studied samples, the most important reductions in microbial load were due to the HHP treatment. However, the incorporation of some encapsulated tomato by-products also reported the additional reduction of this microbial load. For instance, USAE encapsulates seemed to be the most powerful antimicrobials, as they significantly reduced the microbial load compared to the CTRL-HHP or ASE samples after 14, 21, or 28 days of storage for mesophiles, molds, and yeasts, as well as after 14 and 28 days for *Enterobacteriaceae.*

### 3.3. Antioxidant Bioactive Compounds Evolution During Refrigerated Storage

#### 3.3.1. Free Phenolic Content and Antioxidant Capacity

**Figure 4:**
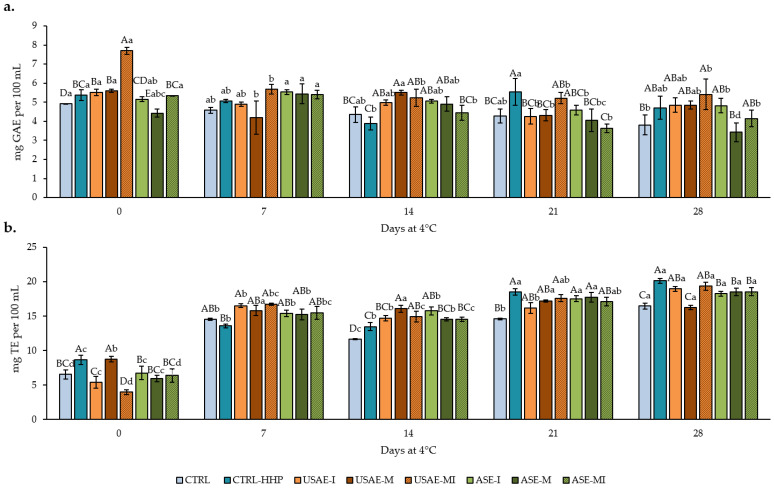
Free phenolic content (**a**) and total antioxidant capacity measured by FRAP (**b**) of seasoned cucumber beverages over 28 days at 4 °C (mean value of three replicates ± SD). Different capital letters denote significant differences among treatments (*p* < 0.05). Different lowercase letters denote significant differences among sampling days (*p* < 0.05). The absence of letters indicates no significant differences (*p* > 0.05) among treatments.

The results of FPC and TAC, presented in Figure 4, demonstrate significant variations influenced by the processing method (HHP), the type of capsule incorporated, and the storage duration. As shown, the FPC values slightly decreased during the refrigerated storage, while the TAC measured by FRAP increased two-fold during the same period in comparison with initial values. This decline in FPC highlights the gradual degradation of phenolic compounds, which are key contributors to antioxidant activity and critical in mitigating oxidative stress during storage. Nevertheless, the increase in the FRAP could be attributed to the presence of ascorbic acid from lemon juice.

Moreover, from a general point of view, USAE samples showed higher values of FPC and TAC, although no great differences were found between them and the CTRL-HHP or ASE samples. In this sense, the highest preservation of phenolic and antioxidant compounds can be observed in all HHP-treated samples, while CTRL samples suffered a general decrease of ~15–16% compared to the remaining formulas for both variables (FPC and TAC), based on average values for all sampling days.

#### 3.3.2. Carotenoid Profile and Content

**Figure 5:**
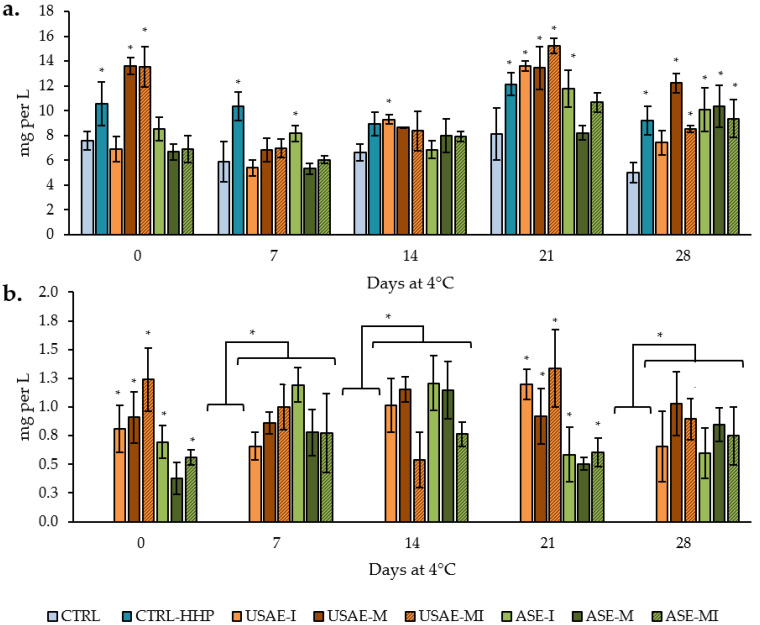
Lutein (**a**) and *β*-carotene (**b**) content in seasoned cucumber beverages over 28 days at 4 °C (mean value of three replicates ± SD). * denotes significant differences *p* < 0.05 compared to CTRL. Figure S3 shows the specific statistical differences among treatments or sampling days.

As the main components of the encapsulated tomato by-products, the carotenoid profile of elaborated samples was quantified, and the obtained results are shown in Figure 5, in which differences among new seasoned cucumber beverages and CTRL samples are shown. Similarly, Figure S3 shows specifically the differences among all studied samples and all sampling days during the refrigerated storage.

Lutein was identified as a common carotenoid in cucumbers, while *β*-carotene was found only in USAE and ASE samples, as it was provided by the different encapsulated tomato by-products. In this sense, the average values obtained for lutein content ranged from 5 to 8 mg per L in CTRL samples, while the fortified samples reached values of 15.2 mg per L of sample. In fact, such increases were higher in USAE samples at days 0 and 21, while no substantial differences were observed at the rest of the sampling times. The same behavior was appreciated with *β*-carotene, which was higher in USAE samples after elaboration and after 21 days, showing values ranging from 0.4 to 1.4 mg per liter, approximately, due to the low concentration of tomato by-products incorporated into each new formula (5 g per liter).

### 3.4. Statistic Correlations

**Figure 6:**
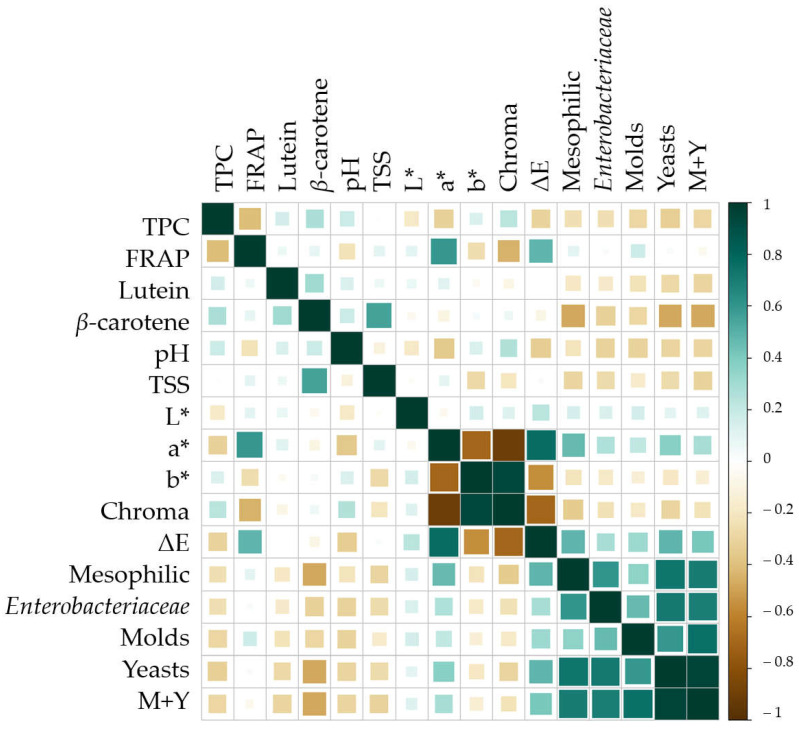
Pearson correlation plot between studied variables of seasoned cucumber beverages over 28 days at 4 °C. TPC: total phenolic content; FRAP: ferric reducing antioxidant power; TSS: total soluble solids; ΔE: color variations; M + Y: molds and yeast. Table S3 shows the correlation coefficients and significance of these correlations.

According to the obtained results, Figure 6 (color plot) and Table S3 (coefficients and *p*-values) show the Pearson correlation among the studied variables and their evolution during the shelf-life study of 28 days at 4 °C. Positive or direct correlations between variables are shown in dark-blue-green, while negative or inverse correlations are shown in brown (Figure 6). The most prominent correlations (*p* < 0.001) show that the presence of phenolic compounds and carotenoids, specifically *β*-carotene, which is the one provided by tomato by-products, is negatively correlated with the microbial growth (mesophilic, enterobacteria, molds, and yeasts). In fact, the reduction of the microbiological load is also positively correlated to a* and ΔE while negatively correlated to b* and chroma, all of which related to the presence of encapsulated tomato by-products (*p* < 0.05). Similarly, the microbial parameters are also negatively correlated to the pH (*p* < 0.001) and TSS (*p* < 0.005), which are linked to the presence of carriers, made of maltodextrin and inulin, that compose the encapsulates of tomato by-products. Also, this correlation could be due to the hydro solubility of maltodextrin and inulin, which, while are being dissolved, are simultaneously releasing more carotenoids to the cucumber beverage, thus increasing its antimicrobial capacity. In addition, all these parameters are related to each other as they are associated with the presence of such encapsulated ingredients, which justifies the positive effect of their incorporation in new formulas such as the seasoned cucumber beverage elaborated in the present work.

## 4. Discussion

The findings of this study demonstrated that high hydrostatic pressure (HHP) technology, combined with various encapsulation methods and carriers (I, M, and the I + M combination), significantly influenced the physicochemical quality of cucumber over 28 days at 4 °C (Table 1 and Table S1, and Figure 2).

As previously described, the mean density of the studied seasoned cucumber beverages was 1090.7 ± 16.4 kg m^−3^ without significant differences among the studied samples. These values agree with previous results obtained by Mohd Rosli et al. [29], who reported 1060 kg m^−3^ for fresh cucumber juice, which is the most similar methodology to comparing our samples. Also, Babajide et al. [30] obtained values of 1020 kg m^−3^ for beverages made of 50% cucumber and 50% pineapple juice, which can justify the little deviation from our obtained data.

Regarding the obtained values of pH, the combined inulin–maltodextrin treatments (ASE-MI and USAE-MI) demonstrated greater stability in maintaining pH, which may be linked to the synergistic effects of these encapsulates of tomato by-products in inhibiting microbial activity and preserving organic compounds. The USAE-M treatment showed the best performance in preserving both color quality and pH stability. These findings suggest that the integration of HHP technology with appropriate encapsulation carriers can effectively enhance the shelf life and physicochemical quality of fresh produce. The results are consistent with previous studies highlighting the efficacy of HHP [31] and encapsulation [12,32] technologies in preserving food quality. Furthermore, our obtained values are comparable to previous data as those reported by El-Saadony et al. [33] in fresh cucumber juices with 0.15% citric acid (4.4), or those from 4.36 to 4.41 reported by Babajide et al. [30] in 50:50 cucumber–pineapple juices, which is similar to our values from 4.60 to 4.87 in seasoned cucumber beverages with 1% lemon juice. Other authors such as Zhang et al. [34] have shown values between 6 and 6.13 for fresh and filtered cucumber juices without any kind of ingredient, which slightly differ from our results of 5.4–5.6 for the blended cucumber juice used in this experiment or the values reported by Liu et al. [35] (5.7), although these little differences can also be explained by the different cultivars used by the authors in China [34,35] and ours in Spain.

The TSS of our raw cucumber beverage was 4.1–4.4 g sugar per 100 mL^−1^, which can be comparable to the values of 3.5 reported by Zhang et al. [34] in their blended cucumbers. In other formulas, Babajide et al.’s study [30] on 50:50 cucumber–pineapple juices reported higher values of total soluble solids (from 7.6 to 8.08 g sugar per 100 mL^−1^), surely due to the use of pineapple in their formula; El-Saadony et al. [33] reported 7.2–7.8 g sugar per 100 mL^−1^ in fresh cucumber juices due to the incorporation of 5.4% of sugar to their juices. These results can justify our 4.7–5.6 g sugar per 100 mL^−1^ of our seasoned cucumber beverage with no added sugar. In fact, the increase in TSS shown in fortified samples has been attributed to the incorporation of the by-product encapsulates, which are made of maltodextrin and inulin as carriers. Maltodextrins with varying dextrose equivalents are frequently used as encapsulating agents because of their excellent water solubility, low viscosity, and clear solutions. As a digestible polymer, maltodextrin allows for the rapid release of the bioactive compounds with antioxidant properties it encapsulates during digestion, making them vulnerable to conditions in the gastrointestinal tract [36]. Inulin, a mildly branched (<5%) fructo-oligosaccharide made up of *β*-(2-1) linked fructose units, is somewhat soluble in water. However, its *β*-(2-1) glycosidic bonds prevent it from being digested by humans, unlike maltodextrin, but it is still broken down by specific microorganisms within the gut [36]. In this sense, their presence in the encapsulates explains the general increase in the TSS in USAE and ASE samples compared to CTRL and CTRL-HHP. Furthermore, the development and the characterization of these encapsulates have been recently published by Cano-Lamadrid et al. [22], and the results already reported a dissolution capacity of 3.2–5.9 g sugar per 100 mL^−1^, which also probes that this increase in TSS is due to the use of carriers as a protective membrane of the encapsulates.

The color coordinates reported can be comparable to those shown by Zhang et al. [34], which were 24.4–27.7 for L*, from −2.2 to −2.7 for a*, and 3.5–5.6 for b*. In fact, the variation in comparison with the data of the present experiment could be due to the 200-mesh filtration carried out by the authors [34]. Also, El-Shaadony et al. [33] showed similar values of color, with 22.8–32.4 for L*, −3.9–0.4 for a*, 10.5–13.6 for b*, and 11.0–14.1 for chroma, which also corroborates our obtained results.

Regarding changes in color parameters (L*, a*, b, chroma, and ∆E), the results indicated that treatments incorporating maltodextrin and the inulin-maltodextrin encapsulates of tomato by-products (USAE-M, ASE-M, USAE-MI, and ASE-MI) exhibited greater stability in preserving the product’s color compared to inulin-based encapsulates (USAE-I and ASE-I) and control samples (CTRL and CTRL-HHP). Specifically, the ASE-MI sample showed the least reduction in the a* parameter, indicating more effective protection of cucumber’s green color. The synergistic effect between inulin and maltodextrin can be attributed to the structural and antioxidant properties of these carriers, which efficiently protect chlorophyll pigments from oxidative degradation in microalgae, thereby enhancing their stability as natural colorants [37].

According to the obtained results for microbial growth, HHP is a technique based on the application of high pressures that effectively prevents the growth of bacteria and the triggering of enzymes responsible for browning or other physicochemical alterations, thereby ensuring food safety for consumption [34,35]. Moreover, in the case of mesophiles and enterobacteria, an additional reduction in microbial was directly related to the presence of encapsulates of tomato by-products (Figure 3), which are rich in carotenoids from tomato by-products [22], specially *β*-carotene, which has a demonstrated antimicrobial capacity [38]. On the other hand, to compare our results of mesophiles (4–5.2 log CFU in CTRL and 2.5–3.5 log CFU in HHP-treated samples), *Enterobacteriaceae* (2.3–2.5 log CFU and 0.7–1.3 log CFU in the rest of the samples), and molds and yeasts (4.1–4.9 log CFU in CTRL and 2.9–3.4 log CFU in HHP samples), previous studies, such as those by Zhang et al. [34], Liu et al. [35], and El-Saadony et al. [33], obtained similar results in fresh cucumber juices. In this sense, El-Saadony et al. [33] showed values from 1.2 to 5 log mesophiles CFU per mL at day 0 and after 6 months of storage in thermally treated cucumber juices. Zhang et al. [34] showed values of 1.7 log CFU mesophiles in 300 MPa-treated cucumber juices and 2.5 log CFU mesophiles in 200 MPa-treated samples, while no yeasts or molds were detected under such conditions. Similarly to this last study, Liu et al. [35] reported no mold or yeast growth during 20 days of refrigerated storage of cucumber beverages treated under 500 MPa HHP for 5 min, while their mesophilic content was 0.3–1 log CFU per mL. In addition, in the case of the juices elaborated by Liu et al. [35], it is important to highlight that only the ultrafiltration performed before HHP treatment was able to reduce 1.5 log CFU mesophiles per mL and 2 log CFU molds and yeasts per mL compared to unfiltered control juices, with 5.2 log CFU mesophiles per mL and 4.5 log CFU per mL for molds and yeasts, values in the same range that the obtained in the present experiment. In this context, although the values obtained in our study did not exceed the safety limits established by international authorities [24,39], the observed differences may be attributable to variations in the procedures employed during the preparation or analysis of the samples.

As described above, FPC and TAC followed different tendencies (Figure 4), while FPC suffered a slight decrease during the storage period, which could be due to their particularly sensitive to decomposition under long storage, temperature, and light exposition variations inside the cold chamber during the shelf-life study [40]. By contrast, the TAC measured by FRAP is increased two- and three-fold during refrigerated storage. This fact could be due to the presence of ascorbic and citric acid provided by lemon juice used in the formula since the antioxidant capacity is not only related to the presence of phenolic compounds but also to the presence of organic acids and carotenoids, whose content is shown in Figure 5. In fact, due to their chemical structure, organic acids have been shown to act as electron donors to other antioxidants such as phenolic compounds or carotenoids, after they have already used their hydroxyl group to avoid the oxidation of the surrounding medium [41], in this case, the cucumber beverage. This can explain the increase in the TAC from the elaboration to the seventh day of refrigerated storage. For instance, the synergistic effect of ascorbic acid and lipid-soluble antioxidants as carotenoids is widely known [42], which has been demonstrated using acerola, rich in vitamin C that serves as an electron donor in the aqueous phase to the carotenoid, lutein, and *β*-carotene that are also present in acerola, as well as in our seasoned beverages. In this sense, this combination of lemon and carotenoids from cucumber (lutein) and encapsulates (*β*-carotene) can justify the exponential increase in the TAC during the refrigerated storage for 28 days (Figure 4b). Indeed, the presence of *β*-carotene in USAE and ASE samples but not in CTRL or CTRL-HHP samples may explain the increased TAC detected in such samples. Furthermore, the encapsulation process can impact the bioavailability and stability of antioxidants, potentially influencing their effectiveness. According to our recently published results about the characterization and development of tomato by-product encapsulates, the FPC and FRAP of USAE and ASE encapsulates ranged from 1.8 to 5.2 g GAE per kg and from 0.5 to 2.4 g TE per kg [22]. In this sense, the inclusion of these ingredients can explain the increase of 1–2 mg GAE per 100 mL and 1.5–2 mg TE per 100 mL of cucumber beverages compared to CTRL and CTRL-HHP, especially after 7 and 14 days of refrigerated storage (Figure 4).

As shown, the main carotenoid identified and quantified in our cucumber beverages was lutein, as also previous authors reported in different cultivars of this vegetable [43,44]. In addition, the kinetic changes during storage of these compounds could be due to the stability of this compound during shelf-life. In this sense, the apparition of *β*-carotene was attributed to the presence of tomato by-products encapsulates, as only was identified in such samples. The stability of carotenoids, including both compounds, was significantly influenced by the type of microparticle used (USAE-I, USAE-M, USAE-MI, ASE-I, ASE-M, and ASE-MI) in seasoned cucumber beverages. Among the tested formulations, mainly USAE samples, and especially those with maltodextrin as a carrier, showed the best protection for lutein against degradation during storage, which could be due to the presence of this carrier in the beverage. In fact, due to the hydro solubility of maltodextrin, these results can be attributed to its ability to form protective microenvironments around carotenoids, minimizing their exposure to oxygen and light, as supported by prior research highlighting the role of maltodextrin in stabilizing bioactive compounds with antioxidant properties during storage [15,36].

On the other hand, the lutein content has been highly protected by HHP treatment in comparison with the CTRL sample, with no treatment, which aligns with findings that HHP preserves carotenoid bioactivity by limiting enzyme-mediated oxidation and isomerization [45]. The results further underline the critical interplay between encapsulation matrix composition, extraction method, and HHP treatment in maintaining carotenoid stability and functionality. This is consistent with the broader literature demonstrating that the combination of advanced encapsulation and non-thermal technologies like HHP can extend shelf life and retain nutritional quality [45,46].

Furthermore, it is important to note that the encapsulates incorporated into the seasoned cucumber beverages were rich in 13-cis-*β*-carotene (~50–75 mg kg^−1^), all-trans-*β*-carotene (~50–75 mg kg^−1^), and primarily lycopene (~200–300 mg kg^−1^) [22]. If 5 g per L of cucumber beverage had been added (1 g of these encapsulates had ~0.1–0.15 mg kg^−1^ *β*-carotene and ~0.2–0.3 mg kg^−1^ lycopene), they should have initially contained ~0.5–0.75 mg *β*-carotene and ~1–1.5 mg of lycopene. However, lycopene was not detected in the cucumber beverages, and the *β*-carotene content was slightly higher than expected with ~0.5–1.5 mg per L of *β*-carotene.

The absence of lycopene in the cucumber beverages enriched with tomato by-products can be attributed to the lipophilic nature of carotenoids, which is why these by-products were encapsulated to ensure their solubility in the aqueous food matrix. The hydrophobic characteristics of carotenoids limit their incorporation and stability in the final product and influence their bioavailability and interaction with other cellular components [6]. Thus, the poor solubility and stability of lycopene in the aqueous cucumber matrix, combined with its affinity for lipophilic environments, likely explain its absence in the enriched beverage.

On the other hand, the absence of lycopene and this slight increase in the *β*-carotene expected could be due to the role of lycopene *β*-cyclase (LCYB) in carotenoid metabolism in horticultural crops, which has been identified in the endocarp of cucumbers. It is involved in the biosynthesis of *γ-, δ-, α*-, and *β*-carotenes derived from lycopene and is also regulated by the *CsaV3_4G022080* and *CsaV3_4G000740* expression genes, highly expressed in *Cucumis sativus* [47]. Moreover, LCYB mediates carbon flow into the two different branches of the carotenoid biosynthesis pathway, to γ- and α-carotenes and to δ- and *β*-carotenes [48,49], which could justify that the lycopene present in the tomato by-products encapsulates could be metabolized into *β*-carotene, that has been identified and quantified in the studied samples. Nevertheless, this theory could be validated in future research using real-time quantitative PCR to confirm the reliability of the RNA sequence data obtained from transcriptome sequencing analysis and to assess the differential gene expression involved in the carotenoid metabolism of our seasoned cucumber beverages, which is one limitation of the present study.

Therefore, the observed correlations among studied variables can be justified by the use of HHP and the presence of antioxidant compounds and *β*-carotene in the tomato by-products encapsulates, which has been demonstrated to be related to the descent of the microbial load (mesophilic, *Enterobacteriaceae*, molds, and yeasts [38]. In addition, the presence of encapsulates, due to their composition of inulin and maltodextrin, also justifies the increase in TSS and the maintenance of color and pH, as physic-chemical variables directly related to microbial growth and preservation [39].

Overall, the study provides evidence that the synergistic use of encapsulation and HHP processing offers a promising strategy to enhance the stability of carotenoids, thereby improving the health benefits and shelf life of vegetable-based beverages. Future investigations should explore carotenoid bioavailability in vivo and under diverse storage conditions to optimize these formulations for commercial applications.

## 5. Conclusions

The ASE- and especially, USAE- encapsulates increased and maintained the TAC and total carotenoid content by the incorporation of *β*-carotene using encapsulates with maltodextrin and inulin carriers, which are also able to protect these molecules during the storage of such ingredients with antioxidant properties before its use as fortifiers in plant-based beverages. Furthermore, the presence of carotenoids, as main antioxidants in tomato by-products, has been able to reduce the microbial load, which was ensured for 28 days at 4 °C by the application of HHP as non-thermal treatment, with significant reductions in mesophilic bacteria, molds, and yeast loads regarding untreated samples. In addition, pH was also reduced while TSS was increased thanks to the incorporation of these ingredients (I and M). While FPC levels declined during storage, total antioxidant capacity increased across all samples, and carotenoid content was kept stable (~9–13 g L^−1^) for the seasoned beverages with antioxidant abilities. These findings underscore the potential of combining HHP technology with appropriate ingredients as encapsulates of by-products to enhance the shelf life, quality, and nutritional value of new seasoned cucumber beverages.

## Figures and Tables

**Table 1:** Physicochemical quality changes in seasoned cucumber beverages stored for 28 days at 4 °C (mean value of three replicates ± SD).

Treatments	Day at 4 °C	pH	TSS	a*	b*	Chroma
**CTRL**	0	4.80 ± 0.00 a	4.50 ± 0.10 C b	−7.65 ± 0.33 BC c	9.12 ± 0.54 ABCD	11.90 ± 0.62 ABC a
	7	4.77 ± 0.06 ab	4.53 ± 0.06 BC ab	−6.3 ± 0.75 A b	8.25 ± 0.54 B	10.38 ± 0.88 B ab
	14	4.63 ± 0.06 C cd	4.73 ± 0.06 E a	−5.95 ± 0.34 A ab	7.38 ± 1.23 D	9.51 ± 0.92 E b
	21	4.60 ± 0.00 B d	4.70 ± 0.10 B ab	−4.89 ± 0.46 A a	7.92 ± 0.53 B	9.31 ± 0.55 B b
	28	4.70 ± 0.00 C bc	4.53 ± 0.06 B ab	−5.51 ± 0.29 ab	8.45 ± 0.57	10.09 ± 0.63 ab
**CTRL-HHP**	0	4.87 ± 0.06 a	4.50 ± 0.10 C ab	−7.31 ± 0.12 AB d	9.17 ± 0.17 ABC a	11.72 ± 0.20 ABC a
	7	4.80 ± 0.00 b	4.43 ± 0.06 C ab	−6.86 ± 0.17 ABC c	8.83 ± 0.18 AB ab	11.18 ± 0.24 AB ab
	14	4.77 ± 0.06 AB b	4.73 ± 0.06 E a	−6.40 ± 0.30 AB b	8.49 ± 0.40 BC b	10.63 ± 0.49 BCD b
	21	4.70 ± 0.00 AB c	4.20 ± 0.35 C b	−6.21 ± 0.07 BCD ab	8.83 ± 0.04 AB ab	10.79 ± 0.06 AB b
	28	4.80 ± 0.00 AB b	4.43 ± 0.23 B ab	−6.01 ± 0.31 a	9.16 ± 0.49 a	10.96 ± 0.58 b
**USAE-I**	0	4.80 ± 0.00 a	5.00 ± 0.00 A b	−6.76 ± 0.03 A c	8.47 ± 0.03 D	10.84 ± 0.00 D
	7	4.80 ± 0.00 a	4.97 ± 0.06 AB b	−6.33 ± 0.16 A b	8.38 ± 0.25 B	10.51 ± 0.29 B
	14	4.73 ± 0.06 ABC b	5.33 ± 0.12 B a	−5.91 ± 0.28 A a	8.29 ± 0.53 BCD	10.18 ± 0.59 CDE
	21	4.63 ± 0.06 B c	5.07 ± 0.06 AB b	−6.01± 0.05 BC ab	8.51 ± 0.18 AB	10.42 ± 0.17 AB
	28	4.70 ± 0.00 C b	5.07 ± 0.06 A b	−6.11 ± 0.30 ab	8.74 ± 0.56	10.66 ± 0.63
**USAE-M**	0	4.83± 0.06	4.70 ± 0.35 BC b	−7.35 ± 0.59 B c	8.95 ± 0.68 BCD a	11.58 ± 0.90 BCD a
	7	4.77 ± 0.06	4.93 ± 0.06 AB ab	−7.14 ± 0.39 BC bc	8.96 ± 0.49 AB a	11.46 ± 0.63 AB a
	14	4.83 ± 0.06 A	4.83 ± 0.06 DE ab	−6.94 ± 0.20 B abc	8.98 ± 0.34 AB a	11.34 ± 0.38 AB a
	21	4.80 ± 0.10 A	4.80 ± 0.00 AB ab	−6.59 ± 0.31 CD ab	8.43 ± 0.46 AB ab	10.70 ± 0.55 AB ab
	28	4.83 ± 0.06 A	5.07 ± 0.15 A a	−6.25 ± 0.42 a	7.87 ± 0.61 b	10.05 ± 0.74 b
**USAE-MI**	0	4.80 ± 0.00 a	5.00 ± 0.10 A b	−7.74 ± 0.20 BC c	9.70 ± 0.35 A a	12.41 ± 0.38 AB a
	7	4.73 ± 0.06 b	5.17 ± 0.06 A b	−6.51 ± 0.27 AB b	8.31 ± 0.37 B ab	10.55 ± 0.46 B b
	14	4.73 ± 0.06 ABC b	5.57 ± 0.06 A a	−6.61 ± 0.38 AB b	7.58 ± 0.30 CD d	10.06 ± 0.04 DE bc
	21	4.60 ± 0.00 B c	5.17 ± 0.35 A b	−5.54 ± 0.23 AB a	7.90 ± 0.42 B cd	9.65 ± 0.47 B c
	28	4.70 ± 0.00 C b	5.07 ± 0.23 A b	−5.80 ± 0.27 a	8.89 ± 0.30 b	10.62 ± 0.38 b
**ASE-I**	0	4.80 ± 0.00 a	5.03 ± 0.12 A	−8.16± 0.29 C d	9.59 ± 0.28 AB a	12.60 ± 0.39 A a
	7	4.77 ± 0.06 ab	4.97 ± 0.06 AB	−7.07 ± 0.12 BC c	8.67 ± 0.23 B ab	11.19 ± 0.26 AB b
	14	4.70 ± 0.00 BC b	5.00 ± 0.10 CD	−6.31 ± 0.13 AB b	8.42 ± 0.43 BCD b	10.52 ± 0.41 BCDE bc
	21	4.60 ± 0.00 B c	4.97 ± 0.06 AB	−5.89 ± 0.19 BC ab	7.86 ± 0.51 B b	9.82 ± 0.51 B c
	28	4.70 ± 0.00 C b	5.00 ± 0.00 A	−580 ± 0.14 a	7.97 ± 0.48 b	9.86 ± 0.36 c
**ASE-M**	0	4.83 ± 0.06 a	5.03 ± 0.06 A	−7.80 ± 0.32 BC c	9.38 ± 0.45 AB	12.20 ± 0.55 ABC a
	7	4.80 ± 0.00 a	4.90 ± 0.30 ABC	−7.31 ± 0.25 C bc	9.14 ± 0.51 AB	11.70 ± 0.55 A ab
	14	4.70 ± 0.00 BC b	5.10 ± 0.10 C	−6.82 ± 0.29 B ab	8.89 ± 0.73 AB	11.21 ± 0.76 BC ab
	21	4.60 ± 0.00 B c	5.00 ± 0.10 AB	−6.55 ± 0.10 CD a	8.75 ± 0.21 AB	10.93 ± 0.22 AB ab
	28	4.70 ± 0.00 C b	5.07 ± 0.06 A	−6.28 ± 0.26 a	8.60 ± 0.52	10.65 ± 0.57 b
**ASE-MI**	0	4.90 ± 0.00 a	4.83 ± 0.06 AB	−7.34 ± 0.36 B b	8.67 ± 0.30 CD	11.36 ± 0.46 CD ab
	7	4.80 ± 0.00 b	4.60 ± 0.69 BC	−7.54 ± 0.59 C b	9.66 ± 1.01 A	12.26 ± 1.16 A a
	14	4.80 ± 0.00 B b	4.97 ± 0.15 CD	−7.74 ± 1.01 C b	9.65 ± 0.23 A	12.39 ± 0.78 A a
	21	4.70 ± 0.00 AB c	5.00 ± 0.10 AB	−6.95 ± 0.60 D ab	9.57 ± 1.05 A	11.83 ± 1.20 A ab
	28	4.73 ± 0.06 BC c	5.07 ± 0.06 A	−6.17 ± 0.20 a	8.47 ± 0.33	10.48 ± 0.37 b

TSS: total soluble solids (g sugar 100 mL^−1^). Different capital letters denote significant differences among treatments (*p* < 0.05). Different lowercase letters denote significant differences among sampling days (*p* < 0.05). The absence of letters indicates no significant differences (*p* > 0.05) among treatments or sampling days.

## Data Availability

Data are contained within the article and Supplementary Materials.

## Supplementary Materials

The following supporting information can be downloaded at: https://www.mdpi.com/article/10.3390/antiox14030354/s1. Table S1: Luminosity (L*) changes in seasoned cucumber beverages over 28 days at 4 °C (mean value of 3 replicates ± sd); Table S2. Detailed ANOVA results for microbial growth in seasoned cucumber beverages over 28 days at 4 °C; Table S3: Pearson correlation matrix between the studied variables of seasoned cucumber beverages over 28 days at 4 °C; Figure S1: HHP treatment conditions using the high-pressure Iso-Lab system (Stansted Fluid Power Ltd., Harlow, UK) for cucumber beverage samples; Figure S2: Chromatography spectrum of lutein (left) and *β*-carotene (right) identified in seasoned cucumber beverages; Figure S3: Lutein (a.) and *β*-carotene (b.) content of seasoned cucumber beverages over 28 days at 4 °C (mean value of 3 replicates ± sd). Different capital letters denote significant differences among treatments (*p* < 0.05). Different lower-case letters denote significant differences among sampling days (*p* < 0.05). No letters denote no differences (*p* > 0.05).
