# Supplementary material for: Boosting Antioxidant Quality in Cucumber Beverages with Encapsulated Tomato Carotenoids
Source: Antioxidants (Basel). 2025 Mar 18;14(3):354. doi: 10.3390/antiox14030354 (PMC11939665; doi:10.3390/antiox14030354)
Supplement: Supplementary file 1 [file antioxidants-14-00354-s001.zip › antioxidants-3517471-supplementary.pdf]

Table S1. Luminosity (L*) changes of seasoned cucumber beverages for 28 days at 4°C (mean value of 3 replicates ± sd).

Treatments	Day at 4°C	L*	Treatments	Day at 4°C	L*
**CTRL**	0	34.75± 0.83 AB	**USAE-MI**	0	35.05 ± 0.82 AB
	7	34.75 ± 1.18 AB		7	34.76 ± 0.73 AB
	14	34.73 ± 1.54 ABC		14	34.47 ± 0.41 BC
	21	35.37 ± 0.64		21	34.95 ± 0.49
	28	36.88 ± 0.50 AB		28	35.44 ± 0.52 AB
**HHP-CTRL**	0	34.78 ± 0.65 AB ab	**ASE-I**	0	35.84 ± 1.05 A
	7	34.91 ± 0.44 AB a		7	34.74 ± 0.70 AB
	14	35.03 ± 0.67 AB a		14	34.31 ± 0.13 BC
	21	34.51 ± 0.22 ab		21	34.93 ± 0.94
	28	33.98 ± 0.23 B b		28	36.22 ± 0.89 A
**USAE-I**	0	34.02 ± 0.04 B bc	**ASE-M**	0	35.25 ± 1.01 AB
	7	33.65 ± 0.39 B c		7	34.84 ± 0.90 AB
	14	33.31 ± 0.95 C c		14	34.43 ± 0.94 BC
	21	34.46 ± 0.43 b		21	35.03 ± 0.49
	28	35.61 ± 0.14 A a		28	35.62 ± 0.74 A
**USAE-M**	0	33.84 ± 1.03 B c	**ASE-MI**	0	34.30 ± 0.81 B b
	7	39.16 ± 0.76 B bc		7	35.55 ± 0.79 A a
	14	34.29 ± 0.51 BC bc		14	35.79 ± 0.17 A a
	21	36.33 ± 0.31 ab		21	35.92 ± 0.73 a
	28	35.99 ± 0.30 A a		28	35.05 ± 0.34 AB

Different capital letters denote significant differences among treatments (p<0.05). Different lower-case letters denote significant differences among sampling days (p<0.05). No letters denote no differences (p>0.05) among treatments or sampling day.

Table S2. Detailed ANOVA results for microbial growth of seasoned cucumber beverages for 28 days at 4°C.

Day	Treatments	Mesophilic	Enterobacteria	Moulds&Yeasts
Initial	CTRL	A c	A a	A d
	CTRL-HHP	B b	C b	B
	USAE-I	BC b	C c	C
	USAE-M	B ab	C b	C
	USAE-MI	B b	B a	C
	ASE-I	BC bc	C b	C
	ASE-M	C c	C b	C
	ASE-MI	BC bc	C b	C b
7	CTRL	A c	b	A e
	CTRL-HHP	C c	a	BC
	USAE-I	BC b	ab	C
	USAE-M	BC bc	ab	C
	USAE-MI	BC b	ab	C
	ASE-I	C c	a	AB
	ASE-M	B b	ab	AB
	ASE-MI	C c	ab	C b
14	CTRL	A d	A ab	A c
	CTRL-HHP	BC b	B ab	B
	USAE-I	BCD b	B c	B
	USAE-M	D c	B ab	B
	USAE-MI	D b	B ab	B
	ASE-I	B ab	B a	B
	ASE-M	CD bc	B ab	B
	ASE-MI	D c	B ab	B ab
21	CTRL	A a	A ab	A a
	CTRL-HHP	BC bc	B ab	B
	USAE-I	CD b	B a	B
	USAE-M	D c	B a	B
	USAE-MI	CD b	AB a	B
	ASE-I	BCD ab	AB a	B
	ASE-M	D c	B ab	B
	ASE-MI	B b	B ab	B a
28	CTRL	A b	A a	A b
	CTRL-HHP	B a	BCD b	B
	USAE-I	CD a	CD bc	B
	USAE-M	D a	D b	B
	USAE-MI	BC a	D b	B
	ASE-I	D a	BCD a	B
	ASE-M	BC a	B a	B
	ASE-MI	BC a	BC a	B b

Different capital letters denote significant differences among treatments ($p<0.05$). Different lower-case letters denote significant differences among sampling days ($p<0.05$). No letters denote no differences ($p>0.05$) among treatments or sampling day.

Table S3. Pearson correlation matrix between studied variables of seasoned cucumber beverages for 28 days at 4°C.

	FRAP	Lutein	β-carotene	pH	TSS	L*	a*	b*	Chroma	ΔE	Mesophilic	*Enterobacteriaceae*	Moulds	Yeasts	M+Y
**TPC**	**-0.4093**	-0.0442	**0.3127**	**0.3374**	0.0147	**-0.1848**	**-0.3135**	0.1374	**0.2361**	**-0.3061**	**-0.2453**	**-0.2402**	**-0.2867**	**-0.3269**	**-0.2840**
	***		***	***		**0.0433**	***		*	***	*	*	**	***	**
**FRAP**		-0.0045	0.0590	**-0.4773**	0.0914	0.0933	**0.5906**	**-0.2561**	**-0.4445**	**0.4834**	0.0982	0.0258	0.1730	0.0248	-0.0511
				***			***	**	***	***					
**Lutein**			0.0152	**-0.2142**	0.1234	-0.1467	-0.0442	-0.0163	0.0131	-0.0352	0.0467	-0.0737	0.0722	0.0022	0.0131
				*											
**β-carotene**				-0.0056	**0.4654**	-0.0774	-0.1111	0.0471	0.0784	-0.1010	**-0.4241**	**-0.3275**	**-0.2458**	**-0.4288**	**-0.4260**
					***						***	***	*	***	***
**pH**					-0.1634	-0.1652	**-0.568**	**0.325**	**0.4702**	**-0.5172**	**-0.2211**	**-0.3225**	**-0.3796**	**-0.3608**	**-0.3222**
							***	***	***	***	*	***	***	***	***
**TSS**						-0.0267	0.0927	**-0.2722**	**-0.2069**	0.0218	**-0.2922**	**-0.2684**	-0.1660	**-0.2681**	**-0.3002**
								**	*		**	**		**	***
**L***							-0.0534	0.1512	0.1182	**0.2320**	0.1442	0.1394	0.1570	0.0953	0.1118
										*					
**a***								**-0.7069**	**-0.9097**	**0.7681**	**0.4665**	**0.2622**	**0.2132**	**0.3723**	0.2826
								***	***	***	***	**	*	***	
**b***									**0.9366**	**-0.5563**	**-0.2127**	**-0.1820**	-0.1364	**-0.1971**	-0.1465
									***	***	*	*		*	
**Chroma**										**-0.7033**	**-0.3498**	**-0.2331**	**-0.1833**	**-0.2943**	**-0.2201**
										***	***	*	*	**	*
**ΔE**											**0.4860**	**0.2843**	**0.3179**	**0.4802**	**0.4121**
											***	**	***	***	***
**Mesophilic**												**0.6015**	**0.3568**	**0.7397**	**0.7048**
												***	***	***	***
Enterobacteriaceae													**0.4689**	**0.7120**	**0.6971**
													***	***	***
**Moulds**														**0.5977**	**0.7515**
														***	***
**Yeasts**															**0.9568**
															***

Correlation coefficients in bold are those significative correlated. * denotes significant correlations p<0.05. ** denotes significant correlations p<0005. *** denotes significant correlations p<0.001.

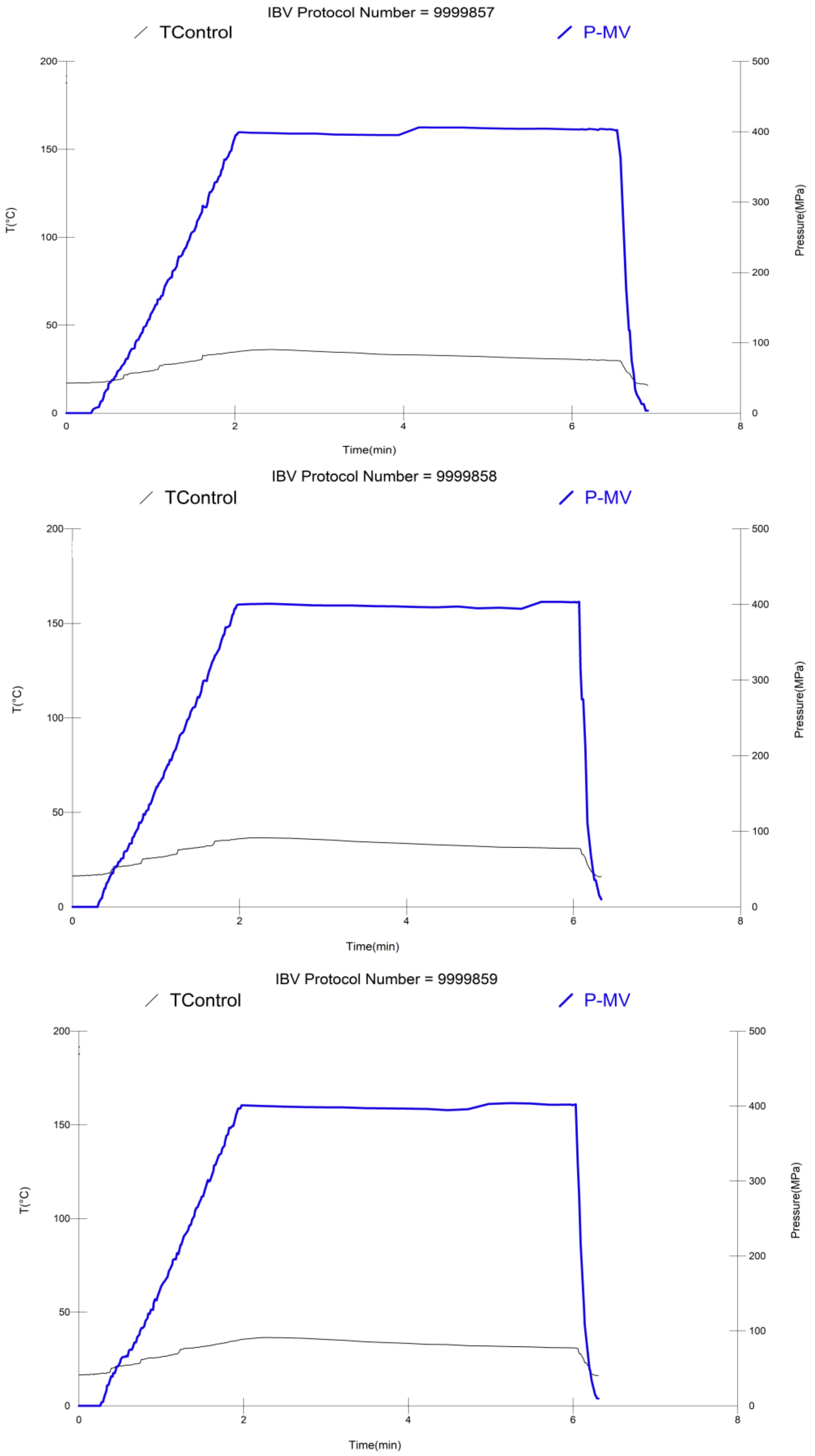

Figure S1. HHP treatment conditions using the high-pressure Iso-Lab system (Stansted Fluid Power Ltd., Harlow, UK) for cucumber beverage samples.

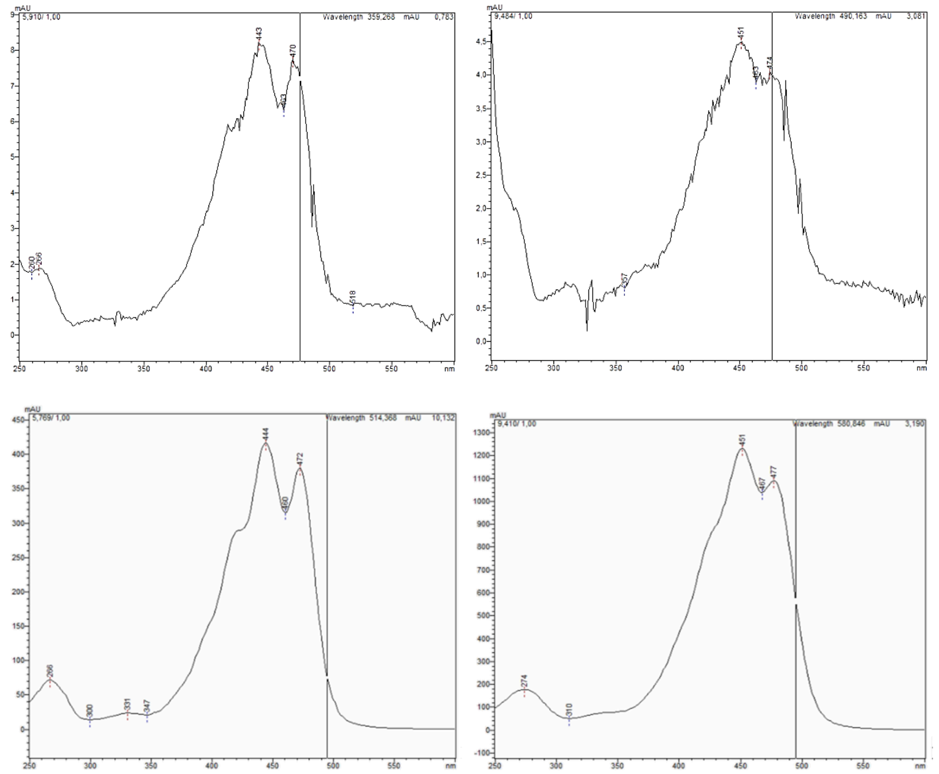

Figure S2. Electromagnetic spectrum of lutein (left) and β-carotene (right) identified in seasoned cucumber beverages (up) and of the standard used for its identification (bottom).

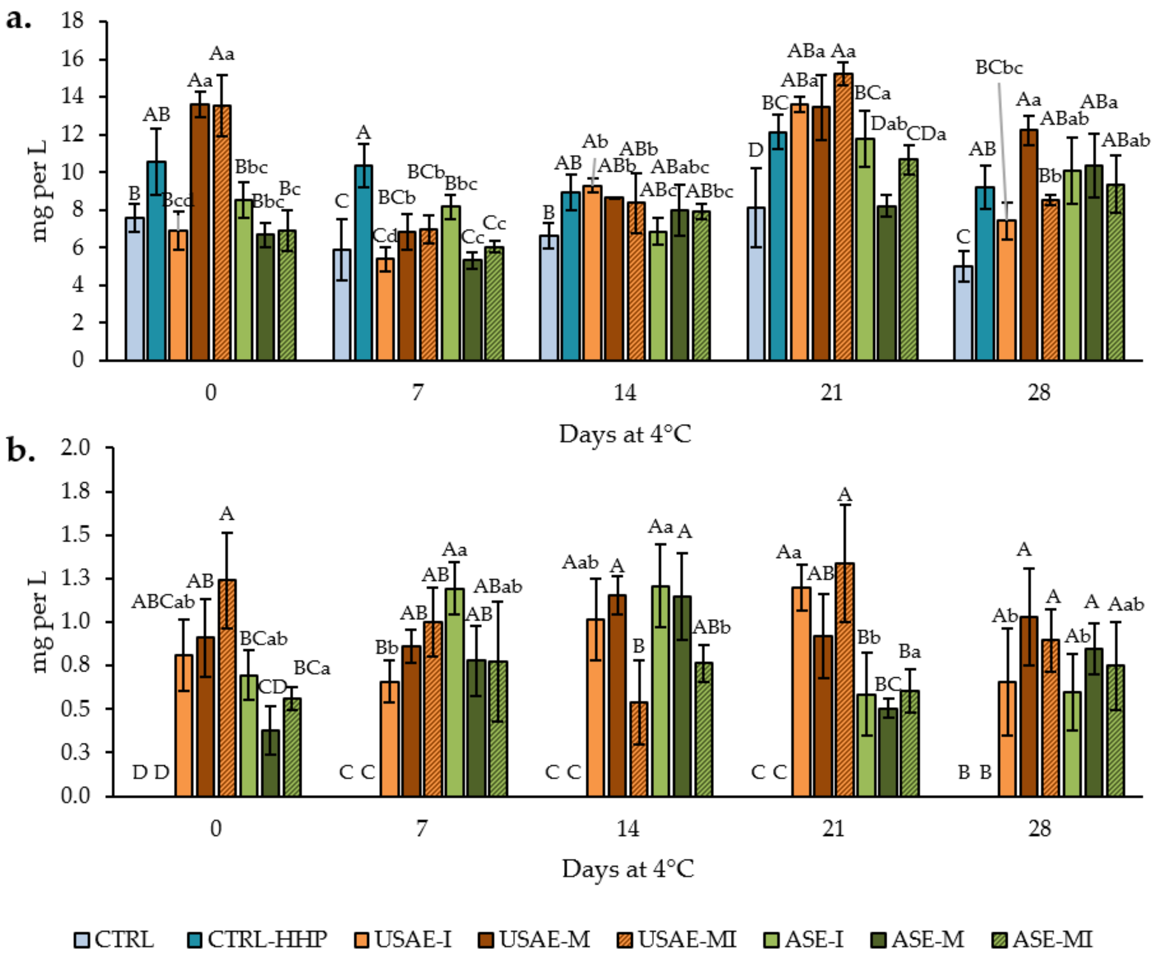

Figure S3. Lutein (a.) and β-carotene (b.) content of seasoned cucumber beverages for 28 days at 4°C (mean value of 3 replicates ± sd). Different capital letters denote significant differences among treatments ($p<0.05$). Different lower-case letters denote significant differences among sampling days ($p<0.05$). No letters denote no differences ($p>0.05$).
